# Changes in UK pre‐schooler's mental health symptoms over the first year of the COVID‐19 pandemic: Data from Co‐SPYCE study

**DOI:** 10.1002/jcv2.12163

**Published:** 2023-04-15

**Authors:** Peter J. Lawrence, Simona Skripkauskaite, Adrienne Shum, Polly Waite, Helen Dodd

**Affiliations:** ^1^ Centre for Innovation in Mental Health School of Psychology University of Southampton Southampton UK; ^2^ Department of Experimental Psychology University of Oxford Oxford UK; ^3^ Department of Psychiatry University of Oxford Oxford UK; ^4^ School of Psychology and Clinical Language Sciences University of Reading Reading UK; ^5^ Children and Young People's Mental Health Research Collaboration Exeter Medical School University of Exeter Exeter UK

**Keywords:** childcare, children, COVID‐19, mental health, pandemic, parents, pre‐school

## Abstract

**Background:**

The COVID‐19 pandemic caused significant disruption to the lives of children and their families. Pre‐school children may have been particularly vulnerable to the effects of the pandemic, with the closure of childcare facilities, playgrounds, playcentres and parent and toddler groups limiting their opportunities for social interaction at a crucial stage of development. Additionally, for parents working from home, caring for pre‐school aged children who require high levels of support and care, was likely challenging. We conducted an intensive longitudinal, but not nationally representative, study to examine trajectories of pre‐schoolers’ mental symptoms in the United Kingdom during the first year of the COVID‐19 pandemic.

**Methods:**

UK‐based parents and carers (*n* = 1520) of pre‐school‐aged children (2–4 years) completed monthly online surveys about their pre‐schoolers’ mental health between April 2020 and March 2021. The survey examined changes in children's emotional symptoms, conduct problems and hyperactivity/inattention.

**Results:**

In our final mixed‐effects models, our predictors (fixed effects) accounted for 5% of the variance in each of conduct problems, emotional symptoms and hyperactivity/inattention symptoms scores, and the combined random and fixed effects accounted for between 64% and 73% of the variance. Pre‐schoolers’ emotional problems and hyperactivity/inattention symptoms declined from April through summer 2020 and then increased again during the autumn and winter 2020/2021 as lockdowns were re‐introduced. Pre‐schoolers who attended childcare showed greater decline in symptom severity than those who did not. Older children, compared to younger, showed greater lability of emotion symptom severity. Attending childcare predicted lower symptom severity across all three domains of conduct problems, emotional symptoms, and hyperactivity/inattention, while the opposite pattern was observed for children whose parent had a mental health problem.

**Conclusions:**

Our findings reinforce the importance of examining pre‐schoolers’ mental health in the context of micro and macro‐level factors. Interventions focussing on family factors such as parent mental health, as well as continued provision of childcare, may have most potential to mitigate the impact of COVID‐19 on young children's mental health.

## Graphical abstract

We examined the trajectories of 1520 UK pre‐school children’s mental health symptoms (conduct problems, emotional problems, and hyperactivity/inattention) in an intensive longitudinal study in the first year of the COVID‐19 pandemic. We found that symptom severity improved following the end of the first national lockdown, and worsened around the later national lockdowns. Crucially, symptom trajectories were moderated by attending childcare (with greater decline in severity for all symptom categories over time for children attending childcare compared to those who did not) and overall symptom severity was moderated by parent mental ill health (symptoms were less severe in all symptom categories for children whose parents did not have a mental health problem than for children whose parents did report a mental health problem).

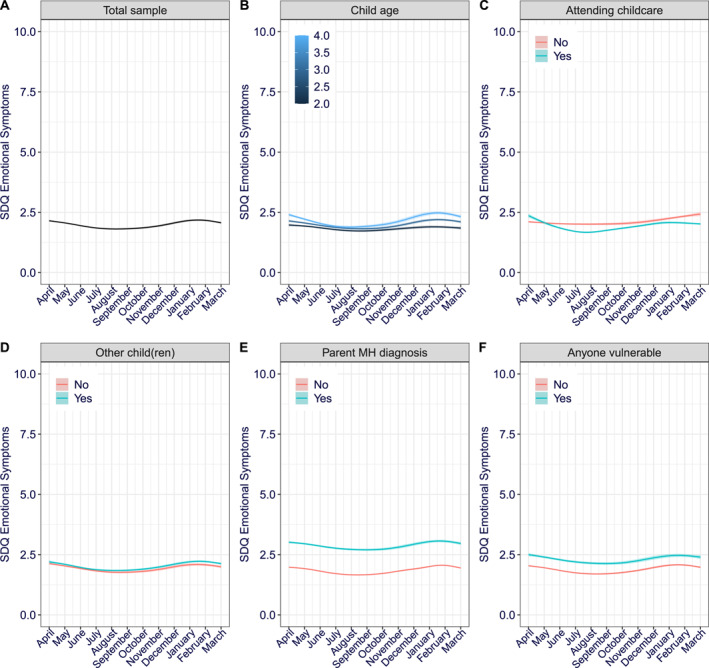

**Key Points**

- Little is known about the mental health of pre‐school aged children in the UK during the Covid‐19 pandemic.
- UK‐based parents and carers (*n* = 1520) of pre‐school‐aged children (2–4 years) completed monthly online surveys about their pre‐schoolers’ mental health over the first year of the pandemic.
- Pre‐schoolers’ emotional problems and hyperactivity/inattention symptoms declined from April through summer 2020 and then increased again during the autumn and winter 2020/2021 as national lockdowns were introduced.
- Attending childcare predicted lower symptom severity across all domains whereas having a parent with a mental health problem predicted higher levels of symptom severity in all domains.
- Intervention focussing on parent mental health and the provision of childcare may mitigate the impact of COVID‐19 on young children's mental health.

## INTRODUCTION

The COVID‐19 pandemic caused significant disruption to the lives of children and their families, via the threat of the virus itself, and the restrictions and pressures placed on them. Pre‐school children (i.e., under the age of 4 years) may have been particularly vulnerable to the effects of the pandemic for a number of reasons. In the UK, the majority of formal childcare closed in March 2020 to all except key‐worker children, with an estimated 90% of children who would usually have attended an early years setting not attending. By June 2020, 83% of children who had been removed from childcare still had not returned (Pascal et al., 2020). Furthermore, social distancing measures prevented children from playing and interacting with their peers, and local facilities where pre‐school children would usually play, such as playgrounds, parent and toddler groups and indoor play centres, were closed (Brown & History of English Lockdown Laws, 2021). Additionally, many parents attempted to work from home while caring for pre‐schoolers, who require high levels of support and care, which may have been particularly challenging. This combination of restrictions on social and physical activity and the increased likelihood of exposure to a range of other risk factors such as parental distress is likely to have had an impact on young children's mental health. Despite this, very little research has focussed on the impact of the pandemic and related restrictions on pre‐school aged children's mental health over time. In this study we therefore evaluate trajectories of mental health symptoms in preschool‐aged children living in the UK from April, 2020 to March, 2021.

Numerous studies have evidenced the negative impact of the pandemic on the mental health of school‐aged children. For example, the NHS Digital survey of English children and adolescents' mental health (Vizard et al., 2020) provided robust and detailed evidence that in the youngest age group examined (5‐ to 10‐year olds), the rate of probable mental health disorder rose from 1 in 11 in 2017 (pre‐pandemic) to 1 in 7 in July, 2020 (in the early months of COVID‐19 related restrictions), with statistically significant increases observed in boys but not girls. Another large study tracking the impact of the pandemic on school‐aged children's mental health in the UK, the Covid‐Supporting Parents, Adolescents and Children during Epidemics (Co‐SPACE) study, collected data from over 8700 families from March 2020 through to March 2021. Results showed that over this period, mental health symptom severity increased during periods of lockdown. Age‐related changes were also found, with younger school‐aged children (4–10 years) reported to have experienced larger changes in the severity of their mental health symptoms (attentional difficulties, behavioural difficulties, emotional difficulties) compared to secondary school‐aged children (11–16 years; Creswell et al., 2021).

Relatively little evidence exists regarding the impact of the pandemic on the mental health of pre‐school aged children and, to our knowledge, no UK‐based study exists. Although cross‐sectional studies have been conducted (e.g. Delvecchio et al., 2022; Foley et al., 2021; Glynn et al., 2021; Jiao et al., 2020; Schmidt et al., 2021; Wang et al., 2021; Zreik et al., 2021), longitudinal research tracking preschool mental health is rare and typically only includes two or three measurement points. Nevertheless, the emerging international literature suggests that, similar to school‐aged children, pre‐school children's mental health also deteriorated during the pandemic and that a range of socio‐demographic factors either attenuated or increased the impact of the pandemic on young children. In Italy, for example, contact with kindergarten attenuated the deterioration of 2–6 year‐old children's mental health from pre‐pandemic to the early stages of the first lockdown (Cantiani et al., 2021). In China, child sex had an impact on 3–6 year‐olds’ anxiety symptoms, with boys faring less well than girls, but only in families whose life had been severely affected by COVID‐19, not in families whose life had been less than severely affected (Ding et al., 2022). Maternal mood moderated the trajectory of 4 year‐olds’ emotional and behavioural problems from pre‐pandemic to the early months of the pandemic in Italy (Frigerio et al., 2022); specifically, children whose mothers reported more severe symptoms of anxiety and depression showed greater increases in a range of emotional and behavioural problems.

These studies highlight the importance of moderating factors, such as parental mental health and contact with childcare, although they do not allow us to draw conclusions regarding the trajectory of UK pre‐schoolers’ mental health symptoms during the pandemic. Children's mental health is affected by a broad range of biological, social and contextual factors and, as a result, we would expect substantial individual differences in response to the pandemic. An individual child's cumulative risk is determined by a range of both risk and protective factors (Vasey et al., 2001). For example, robust risk factors for child mental health difficulties include common mental health difficulties in parents such as anxiety and depression (Lawrence et al., 2019; Tirumalaraju et al., 2020), fearful child temperament and other aspects of the family environment (Hudson et al., 2019; Murray et al., 2009). The pandemic may have affected children's cumulative risk by introducing new risk factors such as parental job loss, exposure to negative media or parent stress, or by exacerbating pre‐existing vulnerabilities or risks, such as a history of a serious medical condition within their social context. In line with this, Prime et al. (2020) offered a conceptual framework for understanding young children's adjustment and development during the COVID‐19 pandemic, highlighting the roles of (a) social disruption, such as confinement and social distancing; (b) caregiver well‐being, such as symptoms of mental ill‐health, (c) pre‐existing vulnerabilities, such as familial serious medical condition and; (d) the wellbeing of the family system and its role in children's resilience to adversity.

The Co‐SPYCE (Covid‐19: Supporting Parents and Young Children in Epidemics) study was established in light of the disruptions caused to UK pre‐schoolers and their families by the COVID‐19 pandemic and tracked UK pre‐schoolers’ mental health over the first year of the pandemic (from April 2020 to March 2021). We use this data to evaluate UK pre‐schoolers’ mental health symptom trajectories during the first year of the Covid‐19 pandemic. We were particularly interested to capture symptom trajectories and to examine micro‐level and, in order to inform future policy decisions, macro‐level contextual factors as potential moderators of trajectories. The paper is unique because of the intensity of longitudinal follow‐up, with monthly assessments conducted over an 11‐month period. This intensive data collection allows nuanced analysis of trajectories over time. This is also to our knowledge the first study to examine how UK pre‐schoolers’ mental health fared during the Covid‐19 pandemic.

In keeping with the findings from UK school‐aged children (Creswell et al., 2021), we predicted that symptom severity would decrease following the end of the first UK lockdown and would then increase as new lockdown restrictions were introduced in late 2020 and early 2021. We also predicted that (a) having (an)other child(ren) at home, and (b) attending a childcare setting, would attenuate the negative impact of the pandemic on symptom trajectories over time (that is, contact with another child, whether at home or in childcare, would buffer the negative impact of isolation from peers), and that each of (c) parent/carer mental health diagnoses, and (d) having a household member vulnerable in light of a history of a serious medical condition, would intensify the negative impact of the pandemic on symptom trajectories. Furthermore, we examined moderator effects of children's age and gender on trajectories but did not hypothesise a specific direction of effect for these.

## METHODS

### Design

The Co‐SPYCE study is an online longitudinal survey composed of a convenience (not nationally representative) sample of UK parents and carers of children aged between 2 and 4 years (Lawrence et al., 2021). The research protocol for the overall Co‐SPYCE study is available via the Open Science Framework (https://osf.io/xyu6c). Ethical approval for the study was provided by the University of Southampton Ethics Committee (ERGO52617).

### Procedure

Participants were recruited through multiple routes, including via partner organisations, networks, charities and schools, print and digital media coverage and social media. Once informed consent was obtained, participants were invited to report on their child in an online Qualtrics survey (www.qualtrics.com/uk). Parents of multi‐child families were asked to identify one ‘index’ child whom they would report on each time. Following completion of the baseline survey, participants were invited back monthly for a follow‐up survey. From December 2021 participants were entered into a monthly prize draw for completion of the survey.

### Participants

Parents and carers (hereafter referred to as ‘parents’) were eligible to take part if they were aged over 18 years, living in the UK and had at least one child aged between 2 and 4 years. In total, 2974 participated (i.e., reached at least the questionnaire section of the survey) in the Co‐SPYCE study between April 17, 2020, and March 31, 2021. The final sample consisted of 1520 parents/carers of children aged, on average, 3 years old at baseline (*M* = 2.94, *SD* = 0.79). These parents completed at least one subscale of the Strengths and Difficulties Questionnaire (Goodman, 1997, 2001) on at least two different occasions and had no missing data on predictor variables (for information on attrition and comparison to national figures see Supplementary Materials: Table S1). On average, they participated 5.06 times (Median = 5, Range: 2–11) with an average gap of 62.56 (Median = 35, Range: 10–335) days between consecutive surveys and provided 5900 observations overall (for participant demographics per number of participations see Supplementary Materials: Table S2). Notably, respondents were predominantly female parents (94%). The majority of the sample was employed (82%), had an average household income of >£16,000 (88%), resided in England (89%), were White (92%) and reported on children who were White (88%).

### Measures

#### Demographics

Parents reported on their child's age, gender, household income, where in the UK they lived, and their family composition (to establish whether there were any other children aged 18 years or younger living in the household). Parents also reported on their own and their family members' histories of serious medical conditions, such as cancer, heart disease and clinically diagnosed chronic physical health conditions.

#### Attending childcare

Parents were asked whether their child had been attending childcare in the last week at the time of completing each survey (yes/no response).

#### Parent/carer mental health diagnosis

Parents were asked whether they had a clinically diagnosed anxiety and/or depressive disorder.

#### Strengths and Difficulties Questionnaire (SDQ) (Goodman, 1997, 2001)

Preschool children's mental health symptoms were measured using the preschool version of the SDQ, a brief behavioural screening questionnaire adapted for preschool‐aged children. The SDQ has been validated in both community and clinical samples and is able to detect psychiatric diagnoses with good sensitivity and specificity (Goodman, 2001; Stone et al., 2010). The preschool SDQ comprises 25 items across five subscales, assessing emotional symptoms, conduct problems, hyperactivity/inattention, peer relationship problems and prosocial behaviour. The focus in this paper is the three subscales that relate to mental health: emotional symptoms, conduct problems and hyperactivity/inattention. Where there was missing data, if at least three of the five subscale items were completed, the person‐mean was imputed. As is a standard requirement for the SDQ, at the first assessment the SDQ asked about symptoms over the last 6 months, and follow‐up assessments asked only about the preceding month. Internal consistency was, on average, acceptable for emotional symptoms (*α* = 0.72), conduct problems (*α* = 0.71), and hyperactivity/inattention symptoms (*α* = 0.76) subscales.

### Data analysis

The statistical analysis plan was pre‐registered via Open Science Framework (OSF; https://osf.io/wxzp7). This plan provides further information on how predictor variables (presence of other children at home, attending childcare, parent/carer mental health diagnoses, and having a household member vulnerable in light of a history of a serious medical condition) were devised from the raw data. Mixed‐effects modelling was conducted in R version 3.6.3 (R Development Core Team, 2015) using the lmer function in lme4 package (Bates et al., 2015). The three outcome variables (SDQ conduct problems, emotional symptoms, and hyperactivity/inattention) were modelled separately. Each model comprised a two‐level structure, where time‐invariant participant information was modelled at the second level with time‐variant repeated measures nested within. This allowed for the random intercept to vary between participants ensuring that the predictions were not confounded by, for example, interindividual biases and that systematic variation in growth trajectories could be assessed (van der Leeden, 1998). The missing data across all models was accounted for by use of maximum likelihood estimation. This approach is preferable to listwise deletion or imputation because it enables parameter estimation with all the available data and maximizes the likelihood of making the observations in the population given the parameters of the model (Hox et al., 2017).

In the first part of the analysis for each outcome, effects of time were explored. More precisely, we modelled polynomial growth modelling of ‘Time’ (i.e., the month when survey was completed coded from 0 = April 2020 to 11 = March 2021) as a linear, quadratic, and cubic trend to determine a pattern of change over‐time. Only the significant, and prior, trends were carried over into further modelling. In the second part of the analysis for each outcome variable, each predictor variable was added to the model (as a main effect) sequentially in a pre‐specified order (child gender, child's grand mean centred age, child attending childcare, presence of other child(ren), parent/carer mental health diagnosis, and anyone in the household vulnerable in light of a history of a serious medical condition), each time observing whether this addition improved model fit. Non‐significant factors (as indicated by model fit) were consequently excluded from further modelling of main effects for that outcome variable. After that, interaction terms were added for each predictor interacting with each time term that was retained in the model as a main effect. Where predictors had not been retained in the model as main effects, the main effect was also reinstated in the model at the same time as the interaction term was evaluated. Interaction terms (and corresponding main effects, if added back in at this stage) were only retained in the model if a significant model improvement was achieved (see Supplementary Materials: Tables S3–S5 for the model selection process).

We used model indices, including AIC, BIC, and χ^2^ log‐likelihood comparisons, to determine whether a particular predictor substantially improved the model. If so, the more constrained model (with the predictor) should have a lower AIC and preferably lower or similar BIC because BIC penalises the model for increased degrees of freedom (Kuha, 2004) than a less constrained model (without the predictor) and produce a log‐likelihood change with χ^2^ test significance of *p* < .05. Furthermore, while our fit indices tell us which is the best of our mixed‐effects models, they do not tell give us a coefficient of determination (*R* ^2^, in fixed effects models). Therefore, we will follow Nakagawa and Schielzeth (2013) and report their *R* ^2^ for mixed models, which comprises two values – the marginal *R* ^2,^, which quantifies how much variance in the outcome is accounted for by fixed effects, or predictor variables, alone, and the conditional *R* ^2^, which quantifies how much variance is accounted for by the fixed and random effects (i.e. varying intercept between participants) together.

## RESULTS

Child age correlated positively with conduct problems (*r* = 0.04, *p* = .001) and emotional symptoms (*r* = 0.09, *p* < .001) across time points, but negatively with hyperactivity/inattention (*r* = −0.06, *p* < .001), although it is important to note that the effect sizes are very small. For participant information on categorical predictor variables, see Table 1. Estimated parameters for final growth curve models across the three outcome variables are presented in Table 2. The model selection process and fit indices are reported in Supplementary Materials (Tables S3–S5).

**TABLE 1:** Means and standard deviations of average SDQ scores across time points, per subscale and predictor category.

	Baseline	Conduct problems		Emotional symptoms		Hyperactivity/Inattention	
	*N* (%)	*M*	SD	*M*	SD	*M*	SD
Total sample	1520 (100%)	2.84	1.98	1.98	1.95	4.74	2.41
Child gender							
Female	755 (50%)	2.71	1.93	2.06	1.97	4.48	2.33
Male	765 (50%)	2.98	2.02	1.90	1.93	5.01	2.46
Child attending childcare							
Yes	228 (15%)	2.69	1.91	1.87	1.91	4.50	2.35
No	1292 (85%)	2.99	2.03	2.09	1.99	4.97	2.44
Presence of other child(ren)							
Yes	499 (33%)	2.90	2.09	1.98	2.00	4.54	2.49
No	1021 (67%)	2.77	1.85	1.99	1.90	4.95	2.29
Parent/carer mental health diagnosis							
Yes	218 (14%)	3.58	2.35	2.89	2.32	5.57	2.58
No	1302 (86%)	2.72	1.89	1.84	1.85	4.61	2.35
Anyone in the household vulnerable in light of a history of a serious medical condition							
Yes	371 (24%)	3.11	2.06	2.31	2.20	4.95	2.35
No	1149 (76%)	2.76	1.94	1.87	1.86	4.67	2.42

**TABLE 2:** Estimated effects and their standard errors (SE) for final models analysis per outcome measure.

	Conduct			Emotional			Hyperactivity/inattention		
	*b*	CI	*p*	*b*	CI	*p*	*b*	CI	*p*
Linear (T1)	−0.25	−0.46 to −0.04	0.018	0.13	−0.10 to 0.36	0.282	−0.08	−0.33 to 0.16	0.503
Quadratic (T2)	0.42	0.22 to 0.62	<0.001	0.41	0.15 to 0.67	0.002	0.38	0.16 to 0.60	0.001
Cubic (T3)				0.12	−0.11 to 0.34	0.310			
Child gender	0.26	0.09 to 0.44	0.002				0.54	0.33 to 0.75	<0.001
Child age				0.17	0.09 to 0.25	<0.001	−0.02	−0.12 to 0.07	0.628
Child attending childcare	−0.17	−0.26 to −0.08	<0.001	−0.11	−0.21 to −0.02	0.021	−0.36	−0.46 to −0.26	<0.001
Presence of other child(ren)	0.17	0.06 to 0.28	0.002	0.07	−0.04 to 0.19	0.201			
Parental mental health diagnosis	0.96	0.71 to 1.21	<0.001	1.00	0.76 to 1.24	<0.001	1.03	0.73 to 1.34	<0.001
Anyone vulnerable (history of medical condition)	0.23	0.03 to 0.44	0.023	0.35	0.16 to 0.54	<0.001			
T1 * child age				0.14	−0.01 to 0.28	0.063	0.22	0.06 to 0.37	0.006
T2 * child age				0.17	0.03 to 0.31	0.021	−0.03	−0.19 to 0.12	0.688
T3 * child age				−0.08	−0.22 to 0.07	0.286			
T1 * child attending childcare	−0.46	−0.74 to −0.17	0.002	−0.31	−0.61 to −0.01	0.042	−0.65	−0.97 to −0.33	<0.001
T2 * child attending childcare	0.07	−0.19 to 0.33	0.594	0.07	−0.26 to 0.40	0.685	0.05	−0.24 to 0.35	0.719
T3 * child attending childcare				−0.60	−0.90 to −0.31	<0.001			

### Conduct problems

**FIGURE 1:**
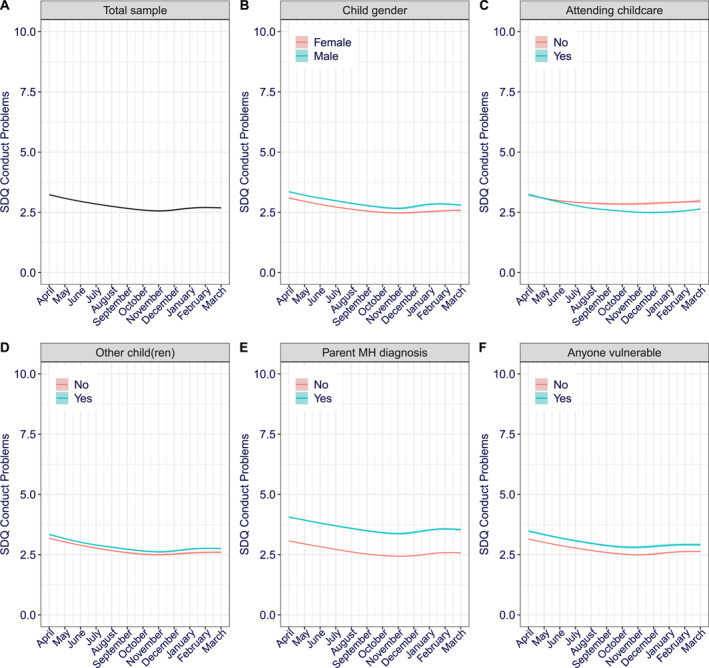
Conduct problems over‐time (A) Total sample, per moderator (B) Child gender (C) Attending childcare (D) Other (children) at home (E) Parent mental health diagnosis (F) Anyone in the household vulnerable. The 95% confidence intervals are shown via shading but note the shaded area is too small to see at this scale without zooming in.

The fixed effects (predictors) alone accounted for 5% of the variance (Nakagawa marginal *R* ^2^ = 0.05) in conduct problems, while the model in full, accounting for fixed and random effects (i.e., varying intercept between participants), accounted for 68% of the variance in conduct problems (Nakagawa conditional *R* ^2^ = 0.68). The growth modelling of conduct problems over‐time identified significant linear and quadratic trends (Table 2); conduct problems generally decreased over the year, but this decrease was more pronounced early on in the pandemic (Figure 1A). Furthermore, the final model included main effects of child gender, presence of other children, parental diagnosis of mental health disorder, whether the child had been attending childcare during the pandemic and whether anyone in the household was vulnerable in light of a history of a serious medical condition (Table 2). Being a boy (Figure 1B), having other children in the household (Figure 1D), having a parent with a diagnosed mental health disorder (Figure 1E) and having someone vulnerable in the household (Figure 1F) all significantly predicted more severe conduct problems. Attending childcare, however, predicted less severe conduct problems than not attending childcare (Figure 1C). An interaction between whether the child had been attending childcare and both linear and quadratic trends of time were also included in the final model, but only the interaction with linear time trend was significant. Specifically, the decrease in conduct problems was more pronounced for children when they had attended childcare during the pandemic than when they had not.

### Emotional symptoms

**FIGURE 2:**
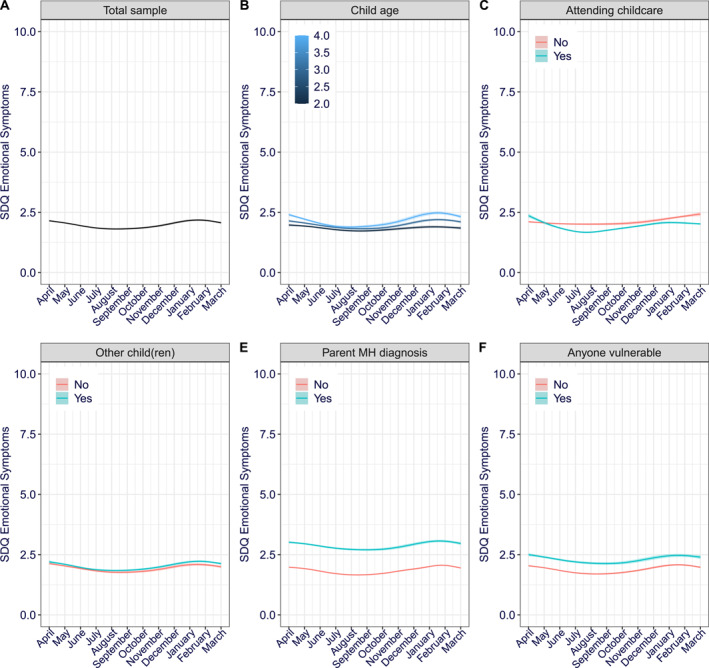
Emotional symptoms over‐time (A) Total sample, per moderator (B) Child age (C) Attending childcare (D) Other (children) at home (E) Parent mental health diagnosis (F) Anyone in the household vulnerable. The 95% confidence intervals are shown via shading but note the shaded area is too small to see at this scale without zooming in.

The fixed effects (predictors) alone accounted for 5% of the variance (Nakagawa marginal *R* ^2^ = 0.05) in emotional symptom scores, while the model in full, accounting for fixed and random effects (i.e., varying intercept between participants) accounted for 64% (Nakagawa conditional *R* ^2^ = 0.64) of the variance in the emotional symptoms scores. Linear, quadratic, and cubic time trends were all included in the final model for emotional symptoms (Table 2). Only the positive quadratic trend remained significant in the final model. In other words, there was no linear change in average severity of emotional symptoms between April 2020 and March 2021. Instead, emotional symptom severity gradually decreased over the summer months, and then began increasing again over the autumn (Figure 2A). Being older (Figure 2B), not attending childcare (Figure 2C), having a parent with a diagnosed mental health disorder (Figure 2E), or anyone in the household classed as clinically vulnerable (Figure 2F) were all associated with higher average emotional symptom severity. Presence of other children in the household was also included in the final emotional symptoms model but it did not significantly predict emotional symptom severity when other predictors were included. Child's age, and whether the child attended childcare, further moderated how emotional symptom severity changed over time (Table 2). Specifically, there was a significant interaction between the child's age and the quadratic term. The quadratic decrease and subsequent increase in emotional symptom severity was more pronounced for older rather than younger children (Figure 2B). There was also a significant interaction between whether the child was attending childcare and both the linear and cubic trends (Figure 2C). In particular, for children attending childcare, emotional symptom severity decreased during the summer, increased during autumn/winter and then decreased again after the winter months. In contrast, emotional symptoms increased over the year for children who were not attending childcare.

### Hyperactivity/inattention symptoms

**FIGURE 3:**
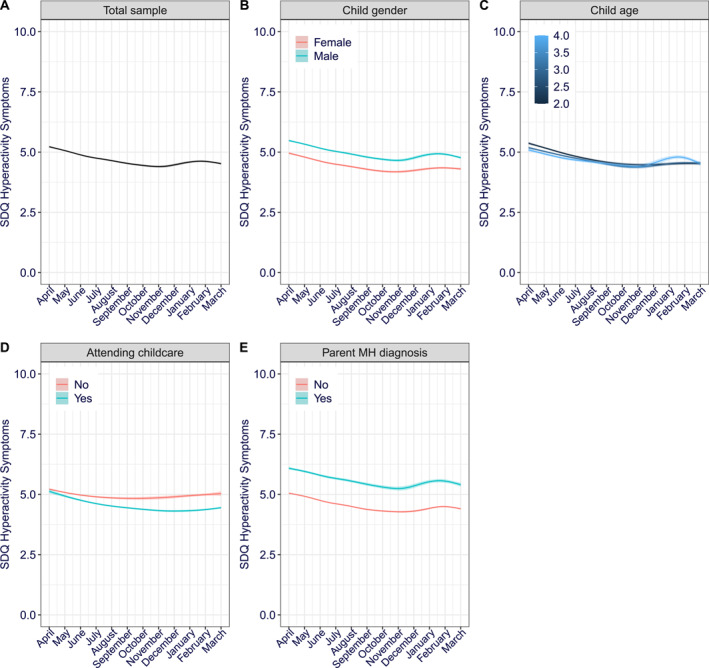
Hyperactivity/inattention symptoms over‐time (A) Total sample, per moderator (B) Child gender (C) Child age (D) Attending childcare (E) Parent mental health diagnosis. The 95% confidence intervals are shown via shading but note the shaded area is too small to see at this scale without zooming in.

Similar to the conduct problems and emotional symptoms models, the fixed effects (predictors) accounted for 5% of the variance in hyperactivity/inattention symptoms scores, while the model in full, accounting for fixed and random effects (i.e., varying intercept between participants), accounted for 73% (Nakagawa conditional *R* ^2^ = 0.73) of the variance in hyperactivity/inattention scores. The final growth modelling for hyperactivity/inattention included linear and quadratic trends, but only the quadratic trend was significant (Table 2). Whilst there was no linear change in average hyperactivity/inattention symptom severity between April 2020 and March 2021, symptom severity gradually decreased over the summer months, and then increased again over the autumn and winter (Figure 3A). The final model included the effects of child gender, child age, whether the child has been attending childcare, and parental diagnosis of a mental health disorder (Table 2). Being a boy (Figure 3B) or having a parent with a diagnosis of a mental health disorder (Figure 3E) significantly predicted more severe hyperactivity/inattention symptoms; whereas attending childcare predicted, on average, less severe hyperactivity/inattention symptoms (Figure 3D). Interactions between, on the one hand, linear and quadratic trends of time and, on the other hand, child age and whether the child had been attending childcare, were also included in the final model (Table 2). Child age significantly moderated the linear effect of time on hyperactivity/inattention symptom severity which, over‐time, increased in older but decreased in younger children. There was also a significant interaction between the linear effect of time and attending childcare. Hyperactivity/inattention decreased with time in children attending childcare, but not in children who were not attending childcare (Figure 3D).

## DISCUSSION

In this longitudinal study we examined trajectories of UK pre‐schoolers’ mental health symptoms during 12 months of the COVID‐19 pandemic. This was a period which included three national lockdowns and closure of childcare services to most children. To our knowledge, this is the first study to intensively track mental health symptoms in young children over a sustained period during the pandemic. The findings are important to identify how young children were affected and inform policy.

We predicted that symptom severity would decrease following the end of the first UK lockdown and would then increase as new lockdown restrictions were introduced in late 2020 and early 2021. The results broadly supported this hypothesis; for emotional problems and hyperactivity/inattention, symptoms initially declined from April through the summer months and then increased again during the autumn and winter of 2020 as lockdowns were reinstated. For conduct problems, a similar pattern was present but the increase during the autumn and winter months was less apparent, and the overall trend was a decrease in symptoms from April 2020 to March 2021.

We also examined micro‐ and macro‐level contextual factors (Foley et al., 2021) as moderators of trajectories of pre‐schoolers’ symptom severity over time. Our hypotheses were partially supported; the only moderators of trajectory over time were attending childcare, which was a significant moderator for conduct problems, emotional symptoms and hyperactivity/inattention; and child age, which significantly moderated changes in emotional symptoms and hyperactivity/inattention. Overall, the final models accounted for 64%–73% of variance in pre‐schoolers’ symptom scores, with 5% of variance (i.e., marginal *R* ^2^ = 0.05) explained by our predictor variables. These effects of the predictors are statistically small but, when considered at a population level, important.

A clear, consistent finding across all three symptom types was that when children were attending childcare, their symptoms showed greater decline (indicating improvements in mental health) over time on average, relative to when children were not attending childcare. For emotional symptoms, there was also an interaction with cubic time, with children attending childcare showing a pattern of more improvement during the summer months followed by more deterioration in the autumn and winter months and then more improvement again after the winter of 2021. Throughout though, children attending childcare had less severe emotional symptoms on average than those who were not attending. The importance of childcare has also been highlighted in previous studies. For example, Cantiani et al. (2021) examined changes in mental health symptoms in pre‐schoolers across two time points (one pre‐pandemic and the second in April 2020) and found that children who had contact with kindergarten showed less decline in their mental health over time, particularly in externalising problems. These findings suggest that the continued social interactions, care and educational experiences offered within formal childcare environments were beneficial for young children, even in the context of a potential higher risk of infection.

For child age as a moderator of patterns over time, there was some indication for emotional symptoms that older preschool‐aged children's symptoms were more labile, particularly around restrictions, with more improvement when restrictions were eased in the summer months of 2020 and more deterioration when restrictions returned in the winter of 2020/2021. Whilst we did not explicitly predict this effect, older children, compared to younger children, might be more aware of restrictions and worried about the consequences of the virus (Vasileva et al., 2021). It is also possible that the older children within our sample were more likely to miss their friends given the significant development that occurs between the ages of 2 and 4 in relation to children's understanding of friendship (Afshordi & Liberman, 2021). It is also possible that older children may have been more likely to articulate their feelings of fear, worry and sadness to parents.

This pattern of more lability in older children was absent for conduct problems and hyperactivity/inattention. For conduct problems, age did not moderate trajectories over time. For hyperactivity/inattention, older children showed less improvement in symptoms over time than younger children, driven primarily by younger children being slightly higher at baseline. This differing starting point may indicate that younger children experienced particularly elevated hyperactivity/inattention symptoms at the start of the pandemic, prior to our initial assessment. Alternatively, it is possible that these are typical age‐related differences. We are unable to differentiate between these explanations within our data but there is quite convincing evidence that young children showed elevated mental health symptoms early in the pandemic. For example, Cantiani et al. (2021) found significant increases in Italian pre‐schooler's mental health symptoms relative to pre‐pandemic levels, when assessed mid to late April 2020. Similar early increases have also been reported in school‐aged children (Waite et al., 2021).

In contrast to our hypotheses, no other factors that we examined acted as moderators of trajectory over time. Several factors did, however, predict the severity of pre‐schoolers’ mental health symptoms, even though they did not affect change over time. For conduct problems, symptoms were more severe when there was at least one other child in the home, when their parent had a diagnosed mental health disorder, when there was a clinically vulnerable person in the household, when the child was not attending childcare, and for boys. For emotional problems, being older, not attending childcare, having a parent with a diagnosed mental health problem and having someone in the household classed as vulnerable were all associated with elevated symptoms. Finally, for hyperactivity/inattention being a boy, having a parent with a diagnosis of a mental health problem and not attending childcare were all associated with elevated symptoms. In addition to attending childcare, which was already highlighted as beneficial for children, the other consistent predictor across all three categories of mental health symptoms was having a parent with a mental health problem (Kiernan & Huerta, 2008). This finding is consistent with a wide range of research demonstrating associations between parent and child mental health (e.g. Amrock & Weitzman, 2014; Hudson et al., 2019; Lawrence et al., 2019) as well as research highlighting parent mental health and distress as a key factor affecting children's mental health during the Covid‐19 pandemic (e.g. Frigerio et al., 2022; Joo & Lee, 2022; Newlove‐Delgado et al., 2021). The finding that boys may be more negatively affected also aligns with research (Ding et al., 2022; Newlove‐Delgado et al., 2021), although this was not consistent across scales in our study, and is in keeping with gender differences in pre‐schoolers outside of the context of the pandemic (Chen, 2010).

### Implications

Older preschool‐aged children, boys, those with a parent who had a mental health disorder, and children not attending childcare were more likely to experience more severe mental health symptoms and to be affected by restrictions. This highlights which children and families are most likely to need support and also has implications for policy. First, the research clearly points to the benefits of attending childcare for pre‐school aged children during the Covid‐19 pandemic and indicates that, at least in relation to mental health, keeping childcare open to young children during health crises will be beneficial. This must be weighed up against the risk to physical health of course, but that was not our focus. Second, children's mental health is closely associated with the mental health of their parents and caregivers. Therefore, to support pre‐school children's mental health it is vital to provide support for parents and carers with their own mental health both within and outside of emergency situations.

### Strengths and limitations

Our study has multiple strengths and limitations. Its strengths include the frequency of follow‐up surveys, allowing us to examine trajectories of symptom severity change; our examination of micro‐ and macro‐level moderators of change, and being the first study to examine UK pre‐schoolers’ mental health during the COVID‐19 pandemic. Regarding limitations, first, our study has no pre‐pandemic data, so we cannot draw inferences about changes in pre‐schoolers’ mental health symptom severity from *before* to *during* the COVID‐19 pandemic. Note though that research including pre‐pandemic baselines indicates that pre‐school children's mental health worsened quickly (Cantiani et al., 2021). Thus, our start point is likely to reflect these already elevated levels of mental health problems. Second, our convenience sample, while drawn from around the UK, is not representative of the UK population of pre‐schoolers; it has an over‐representation of affluent families identifying as white which prevented us from examining the role of ethnicity and income as predictors. There is evidence that older children growing up in lower income households were more likely to have high mental health symptoms during the pandemic (Guzman Holst et al., 2023). Further, adults from ethnic minorities showed greater increases in mental health problems during the pandemic, relative to White adults (Proto & Quintana‐Domeque, 2021). Thus, the overrepresentation of affluent White families in our study means that we are likely to have underestimated the severity of mental health problems across this period of the pandemic. Third, given the young age of the children, all data were parent‐report, which can be a source of bias (Najman et al., 2000). Whilst interrater bias (Hoyt, 2000) was explicitly incorporated in the current analysis via nested structure of the mixed‐effect modelling (van der Leeden, 1998). It should be noted though that parents tend to report higher symptom scores in comparison to, for example, teachers (Elberling et al., 2010). Furthermore, it remains possible that changes in parent mental health over time may have led to biased reporting of their child's mental health. The findings could therefore have been different if a multi‐informant approach had been feasible. Fourth, we used a binary index for parent mental health (lifetime diagnosis: presence or absence), childcare attendance (yes/no) and presence of a sibling. In each of these examples, further information (e.g. mental health symptom severity, time in childcare, type of childcare, age of siblings) may have provided more nuanced insight into associations with pre‐schooler mental health. However, close examination of these factors was outside the scope of the present paper. These topics may be of interest for future research.

## CONCLUSIONS

Findings from this longitudinal study with a non‐representative sample of 1520 parents reinforces the importance of examining pre‐schoolers’ mental health in the context of micro‐ and macro‐level factors; and that interventions focussing on family factors such as parent mental health, as well as continued provision of childcare, might have most potential to mitigate the impact of COVID‐19 on young children's mental health.

## AUTHOR CONTRIBUTIONS

**Peter J. Lawrence**: Conceptualization, Funding acquisition, Investigation, Methodology, Project administration, Resources, Supervision, Writing – original draft, Writing – review & editing. **Simona Skripkauskaite**: Conceptualization, Data curation, Formal analysis, Investigation, Methodology, Project administration, Supervision, Visualization, Writing – original draft, Writing – review & editing. **Adrienne Shum**: Project administration, Resources, Visualization, Writing – review & editing. **Polly Waite**: Conceptualization, Data curation, Funding acquisition, Investigation, Methodology, Supervision, Project administration, Writing – original draft, Writing – review & editing. **Helen Dodd**: Conceptualization, Funding acquisition, Investigation, Methodology, Project administration, Supervision, Writing – original draft, Writing – review & editing.

## CONFLICT OF INTEREST STATEMENT

The authors declare no conflicts of interest.

### OPEN RESEARCH BADGES

This article has been awarded Open Materials, Preregistered badges. All materials and data are publicly accessible via the Open Science Framework at (https://osf.io/xyu6c and https://osf.io/wxzp7). Learn more about the Open Practices badges from the Center for Open Science: https://osf.io/tvyxz/wiki.

## Ethical considerations

The authors obtained approval for this study from the University of Southampton Ethics Committee (ERGO52617). All participants provided informed consent to participate in the study. No material was reproduced from other sources.

## Supporting information

Supplementary Material S1

## Data Availability

The research materials can be accessed by contacting the corresponding author. We are in the process of making the data open access via the UK data service.
