# Supplementary material for: Changes in UK pre‐schooler's mental health symptoms over the first year of the COVID‐19 pandemic: Data from Co‐SPYCE study
Source: JCPP Adv. 2023 Apr 15;3(2):e12163. doi: 10.1002/jcv2.12163 (PMC10519734; doi:10.1002/jcv2.12163)
Supplement: Supplementary file 1 — Supplementary Material S1 [file JCV2-3-e12163-s001.docx]

Supplementary Materials

**Table S1**

Demographic characteristics (at Baseline) of the final sample in comparison to the excluded participants and national figures

|  | Initial sample | Attrition | | | Final sample | National figures |
| --- | --- | --- | --- | --- | --- | --- |
|  |  | Missing outcomes | Missing predictors | Single participation |  |  |
|  | (*N*=2974) | (*N*=460) | (*N*=141) | (*N*=942) | (*N*=1520) |  |
| Parent/carer gender |  |  |  |  |  |  |
| Female | 2720 (91%) | 372 (81%) | 117 (83%) | 880 (93%) | 1428 (94%) | 50.6%^a^ |
| Male | 204 (7%) | 43 (9%) | 14 (10%) | 60 (6%) | 91 (6%) | 49.4%^a^ |
| Other/Unknown | 50 (2%) | 45 (10%) | 10 (7%) | 2 (0%) | 1 (0%) | - |
| Relationship to child |  |  |  |  |  |  |
| Parent | 2797 (94%) | 295 (64%) | 107 (76%) | 939 (100%) | 1510 (99%) | - |
| Grandparent | 17 (1%) | 10 (2%) | 1 (1%) | 1 (0%) | 7 (1%) | - |
| Step-parent | 5 (0%) | 2 (0%) | 1 (1%) | 0 (0%) | 2 (0%) | - |
| Other/Unknown | 155 (5%) | 153 (33%) | 32 (23%) | 2 (0%) | 1 (0%) | - |
| Employment status |  |  |  |  |  |  |
| Education | 70 (2%) | 9 (2%) | 3 (2%) | 25 (3%) | 33 (2%) | 0.1%^b^ |
| Homemaker/full-time parent | 335 (11%) | 22 (5%) | 13 (9%) | 113 (12%) | 195 (13%) | 0.3%^b^ |
| Self employed | 172 (6%) | 18 (4%) | 5 (4%) | 60 (6%) | 91 (6%) | 14.1%^b^ |
| Working full time | 1070 (36%) | 84 (18%) | 48 (34%) | 374 (40%) | 578 (38%) | 61.2%^b^ |
| Working part time | 1010 (34%) | 67 (15%) | 33 (23%) | 341 (36%) | 583 (38%) | 20.2%^b^ |
| Unemployed/Other | 256 (9%) | 4 (1%) | 4 (3%) | 24 (3%) | 29 (2%) | 4.1%^b^ |
| Country |  |  |  |  |  |  |
| England | 2614 (88%) | 367 (80%) | 117 (83%) | 849 (90%) | 1354 (89%) | 84.3%^c^ |
| Northern Ireland | 25 (1%) | 4 (1%) | 4 (3%) | 5 (1%) | 12 (1%) | 2.8%^c^ |
| Scotland | 213 (7%) | 38 (8%) | 10 (7%) | 58 (6%) | 115 (8%) | 8.2%^c^ |
| Wales | 83 (3%) | 12 (3%) | 3 (2%) | 30 (3%) | 39 (3%) | 4.7%^c^ |
| Missing | 39 (1%) | 39 (8%) | 7 (5%) | 0 (0%) | 0 (0%) | - |
| Parent/carer ethnicity |  |  |  |  |  |  |
| White: British, Irish, other | 2500 (84%) | 236 (51%) | 85 (60%) | 831 (88%) | 1401 (92%) | 81.7%^d^ |
| Other ethnic backgrounds | 251 (8%) | 55 (12%) | 14 (10%) | 90 (10%) | 94 (6%) | 18.3% ^d^ |
| Missing | 223 (8%) | 169 (37%) | 42 (30%) | 21 (2%) | 25 (2%) | - |
| Child ethnicity |  |  |  |  |  |  |
| White: British, Irish, other | 2390 (80%) | 220 (48%) | 83 (59%) | 797 (85%) | 1340 (88%) | - |
| Other ethnic backgrounds | 263 (9%) | 59 (13%) | 11 (8%) | 102 (11%) | 95 (6%) | - |
| Missing | 321 (11%) | 181 (40%) | 47 (33%) | 43 (5%) | 85 (6%) | - |
| Household income |  |  |  |  |  |  |
| Less than £16,000 a year | 204 (7%) | 16 (4%) | 14 (10%) | 94 (10%) | 83 (6%) | 12.8%^e^ |
| Less than £30,000 a year | 340 (11%) | 29 (6%) | 20 (14%) | 124 (13%) | 173 (11%) | 36.1%^e^ |
| More than £30,000 a year | 1986 (67%) | 131 (29%) | 61 (43%) | 657 (70%) | 1161 (76%) | 51.1%^e^ |
| Missing | 444 (15%) | 284 (62%) | 46 (33%) | 67 (7%) | 103 (7%) | - |

*Notes.* ^a^ Census 2011 data by gender ^1^; ^b^ Proportion of economically active UK population in Mar-May 2020 ^2^; ^c^ 2020 UK population by region ^3^; ^d^ Census 2021 data by ethnic group ^4^; ^e^ Proportion of UK households in 2020 with an average household income < £16,000 p.a. (Office for National Statistics, 2021).

**Table S2**

Demographic characteristics of the final sample (at Baseline) per number of completed surveys

|  | Two | Three | Four | Five | Six | Seven | Eight | Nine | Ten | Eleven |
| --- | --- | --- | --- | --- | --- | --- | --- | --- | --- | --- |
|  | (*N*=543) | (*N*=312) | (*N*=202) | (*N*=164) | (*N*=95) | (*N*=76) | (*N*=56) | (*N*=26) | (*N*=40) | (*N*=6) |
| Parent/carer gender |  |  |  |  |  |  |  |  |  |  |
| Female | 510 (94%) | 294 (94%) | 186 (92%) | 155 (95%) | 92 (97%) | 68 (90%) | 52 (93%) | 26 (100%) | 39 (98%) | 6 (100%) |
| Male | 33 (6%) | 17 (5%) | 16 (8%) | 9 (6%) | 3 (3%) | 8 (11%) | 4 (7%) | 0 (0%) | 1 (3%) | 0 (0%) |
| Other/Unknown | 0 (0%) | 1 (0%) | 0 (0%) | 0 (0%) | 0 (0%) | 0 (0%) | 0 (0%) | 0 (0%) | 0 (0%) | 0 (0%) |
| Relationship to child |  |  |  |  |  |  |  |  |  |  |
| Parent | 542 (100%) | 309 (99%) | 200 (99%) | 163 (99%) | 93 (98%) | 75 (99%) | 56 (100%) | 26 (100%) | 40 (100%) | 6 (100%) |
| Grandparent | 1 (0%) | 2 (1%) | 1 (1%) | 1 (1%) | 1 (1%) | 1 (1%) | 0 (0%) | 0 (0%) | 0 (0%) | 0 (0%) |
| Step-parent | 0 (0%) | 0 (0%) | 1 (1%) | 0 (0%) | 1 (1%) | 0 (0%) | 0 (0%) | 0 (0%) | 0 (0%) | 0 (0%) |
| Other/Unknown | 0 (0%) | 1 (0%) | 0 (0%) | 0 (0%) | 0 (0%) | 0 (0%) | 0 (0%) | 0 (0%) | 0 (0%) | 0 (0%) |
| Employment status |  |  |  |  |  |  |  |  |  |  |
| Education | 11 (2%) | 6 (2%) | 5 (3%) | 3 (2%) | 2 (2%) | 2 (3%) | 0 (0%) | 2 (8%) | 2 (5%) | 0 (0%) |
| Homemaker/full-time parent | 68 (13%) | 41 (13%) | 24 (12%) | 25 (15%) | 10 (11%) | 12 (16%) | 6 (11%) | 4 (15%) | 4 (10%) | 1 (17%) |
| Self employed | 37 (7%) | 19 (6%) | 12 (6%) | 5 (3%) | 7 (7%) | 4 (5%) | 3 (5%) | 2 (8%) | 1 (3%) | 1 (17%) |
| Working full time | 213 (39%) | 113 (36%) | 90 (45%) | 63 (38%) | 28 (30%) | 23 (30%) | 23 (41%) | 6 (23%) | 17 (43%) | 2 (33%) |
| Working part time | 192 (35%) | 128 (41%) | 67 (33%) | 67 (41%) | 46 (48%) | 31 (41%) | 22 (39%) | 12 (46%) | 16 (40%) | 2 (33%) |
| Unemployed/Other | 17 (3%) | 1 (0%) | 3 (2%) | 1 (1%) | 2 (2%) | 3 (4%) | 2 (4%) | 0 (0%) | 0 (0%) | 0 (0%) |
| Country |  |  |  |  |  |  |  |  |  |  |
| England | 483 (89%) | 278 (89%) | 184 (91%) | 141 (86%) | 79 (83%) | 72 (95%) | 51 (91%) | 25 (96%) | 35 (88%) | 6 (100%) |
| Northern Ireland | 5 (1%) | 0 (0%) | 1 (1%) | 2 (1%) | 3 (3%) | 1 (1%) | 0 (0%) | 0 (0%) | 0 (0%) | 0 (0%) |
| Scotland | 33 (6%) | 29 (9%) | 11 (5%) | 18 (11%) | 10 (11%) | 3 (4%) | 5 (9%) | 1 (4%) | 5 (13%) | 0 (0%) |
| Wales | 22 (4%) | 5 (2%) | 6 (3%) | 3 (2%) | 3 (3%) | 0 (0%) | 0 (0%) | 0 (0%) | 0 (0%) | 0 (0%) |
| Parent/carer ethnicity |  |  |  |  |  |  |  |  |  |  |
| White: British, Irish, other | 492 (91%) | 288 (92%) | 185 (92%) | 157 (96%) | 90 (95%) | 69 (91%) | 53 (95%) | 25 (96%) | 36 (90%) | 6 (100%) |
| Other ethnic backgrounds | 35 (6%) | 18 (6%) | 16 (8%) | 6 (4%) | 4 (4%) | 7 (9%) | 3 (5%) | 1 (4%) | 4 (10%) | 0 (0%) |
| Missing | 16 (3%) | 6 (2%) | 1 (1%) | 1 (1%) | 1 (1%) | 0 (0%) | 0 (0%) | 0 (0%) | 0 (0%) | 0 (0%) |
| Child ethnicity |  |  |  |  |  |  |  |  |  |  |
| White: British, Irish, other | 470 (87%) | 275 (88%) | 181 (90%) | 147 (90%) | 86 (91%) | 67 (88%) | 51 (91%) | 22 (85%) | 35 (88%) | 6 (100%) |
| Other ethnic backgrounds | 40 (7%) | 21 (7%) | 10 (5%) | 7 (4%) | 4 (4%) | 6 (8%) | 4 (7%) | 2 (8%) | 1 (3%) | 0 (0%) |
| Missing | 33 (6%) | 16 (5%) | 11 (5%) | 10 (6%) | 5 (5%) | 3 (4%) | 1 (2%) | 2 (8%) | 4 (10%) | 0 (0%) |
| Household income |  |  |  |  |  |  |  |  |  |  |
| Less than £16,000 a year | 33 (6%) | 16 (5%) | 13 (6%) | 7 (4%) | 4 (4%) | 3 (4%) | 2 (4%) | 2 (8%) | 2 (5%) | 1 (17%) |
| Less than £30,000 a year | 54 (10%) | 36 (12%) | 23 (11%) | 24 (15%) | 14 (15%) | 6 (8%) | 8 (14%) | 7 (27%) | 0 (0%) | 1 (17%) |
| More than £30,000 a year | 419 (77%) | 239 (77%) | 153 (76%) | 126 (77%) | 71 (75%) | 59 (78%) | 43 (77%) | 15 (58%) | 32 (80%) | 4 (67%) |
| Missing | 37 (7%) | 21 (7%) | 13 (6%) | 7 (4%) | 6 (6%) | 8 (11%) | 3 (5%) | 2 (8%) | 6 (15%) | 0 (0%) |

**Table S3**

Model selection and fit indices for conduct problems

|  |  |  | *df* | *AIC* | *BIC* | *Chi2* | *p* |
| --- | --- | --- | --- | --- | --- | --- | --- |
| 1) | Growth curve modelling | |  |  |  |  |  |
|  | M0: | intercept only | 3 | 21515 | 21535 |  |  |
|  | M1: | M0 + Linear time | 4 | 21436 | 21462 | 81.69 | <.001 |
|  | M2: | M1 + Quadratic time | 5 | 21392 | 21425 | 45.81 | <.001 |
|  | M3: | M2 + Cubic time | 6 | 21394 | 21434 | <0.01 | .978 |
| 2) | Selection of main effects | |  |  |  |  |  |
|  | M4: | M2 + Child gender | 6 | 21385 | 21425 | 8.51 | .004 |
|  | M5: | M4 + Child age | 7 | 21386 | 21432 | 1.80 | .180 |
|  | M6: | M5 + Child attending care | 8 | 21375 | 21429 | 12.19 | <.001 |
|  | M7: | M6 + Other child(ren) | 9 | 21369 | 21429 | 8.02 | .005 |
|  | M8: | M7 + Parental MH | 10 | 21308 | 21375 | 63.04 | <.001 |
|  | M9: | M8 + Anyone vulnerable (medical condition) | 11 | 21305 | 21379 | 5.21 | .022 |
| 3) | Selection of interaction effects | |  |  |  |  |  |
|  | M10: | M9 + Child gender * (Linear & Quadratic time) | 13 | 21308 | 21395 | 0.79 | .673 |
|  | M11: | M9 + Child age *(Linear & Quadratic time) | 13 | 21306 | 21393 | 3.01 | .222 |
|  | M12: | M9 + Child attending care * (Linear & Quadratic time) | 13 | 21299 | 21386 | 9.84 | .007 |
|  | M13: | M12 + Other child(ren) * (Linear & Quadratic time) | 15 | 21302 | 21402 | 1.35 | .509 |
|  | M14: | M12 + Parental MH * (Linear & Quadratic time) | 15 | 21300 | 21400 | 3.31 | .191 |
|  | M15: | M12 + Anyone vulnerable (medical condition) * (Linear & Quadratic time) | 15 | 21301 | 21401 | 2.21 | .331 |
| 4) | Final model [M16] | |  |  |  |  |  |
|  | M0 + Linear time + Quadratic time + Child gender + Child attending care + Other child(ren) + Parental MH + Anyone vulnerable (medical condition) + Child attending care * (Linear & Quadratic time) | | 12 | 21299 | 21379 |  |  |

*Note.* Final model of the main analysis includes all effects identified as improving model fit in the previous steps of the main analysis.

**Table S4**

Model selection and fit indices for emotional symptoms

|  |  |  | *df* | *AIC* | *BIC* | *Chi2* | *p* |
| --- | --- | --- | --- | --- | --- | --- | --- |
| 1) | Growth curve modelling | |  |  |  |  |  |
|  | M0: | intercept only | 3 | 21615 | 21635 |  |  |
|  | M1: | M0 + Linear time | 4 | 21614 | 21641 | 3.25 | .071 |
|  | M2: | M1 + Quadratic time | 5 | 21576 | 21610 | 39.73 | <.001 |
|  | M3: | M2 + Cubic time | 6 | 21571 | 21611 | 7.51 | .006 |
| 2) | Selection of main effects | |  |  |  |  |  |
|  | M4: | M3 + Child gender | 7 | 21571 | 21617 | 2.32 | .128 |
|  | M5: | M4 + Child age | 8 | 21558 | 21611 | 14.61 | <.001 |
|  | M6: | M5 + Child attending care | 9 | 21541 | 21601 | 19.01 | <.001 |
|  | M7: | M6 + Other child(ren) | 10 | 21542 | 21609 | 1.08 | .300 |
|  | M8: | M7 + Parental MH | 11 | 21469 | 21542 | 75.12 | <.001 |
|  | M9: | M8 + Anyone vulnerable (medical condition) | 12 | 21459 | 21539 | 12.22 | <.001 |
| 3) | Selection of interaction effects | |  |  |  |  |  |
|  | M10: | M9 + Child gender * (Linear & Quadratic & Cubic time) | 15 | 21463 | 21563 | 1.45 | .694 |
|  | M11: | M9 + Child age * (Linear & Quadratic & Cubic time) | 15 | 21454 | 21554 | 10.50 | .015 |
|  | M12: | M11 + Child attending care * (Linear & Quadratic & Cubic time) | 18 | 21441 | 21561 | 19.46 | <.001 |
|  | M13: | M12 + Other child(ren) * (Linear & Quadratic & Cubic time) | 21 | 21444 | 21584 | 2.68 | .443 |
|  | M14: | M12 + Parental MH * (Linear & Quadratic & Cubic time) | 21 | 21446 | 21587 | 0.29 | .961 |
|  | M15: | M12 + Anyone vulnerable (medical condition) * (Linear & Quadratic & Cubic time) | 21 | 21441 | 21582 | 5.34 | .149 |
| 4) | Final model [M16] | |  |  |  |  |  |
|  | M0 + Linear time + Quadratic time + Cubic time + Child age + Child attending care + Other child(ren) + Parental MH + Anyone vulnerable (medical condition) + Child age * (Linear & Quadratic & Cubic time) + Child attending care * (Linear & Quadratic & Cubic time) | | 17 | 21441 | 21555 |  |  |

*Note.* Final model of the main analysis includes all effects identified as improving model fit in the previous steps of the main analysis.

**Table S5**

Model selection and fit indices for hyperactivity/inattention

|  |  |  | *df* | *AIC* | *BIC* | *Chi2* | *p* |
| --- | --- | --- | --- | --- | --- | --- | --- |
| 1) | Growth curve modelling | |  |  |  |  |  |
|  | M0: | intercept only | 3 | 23162 | 23182 |  |  |
|  | M1: | M0 + Linear time | 4 | 23065 | 23091 | 99.16 | <.001 |
|  | M2: | M1 + Quadratic time | 5 | 23015 | 23049 | 51.34 | <.001 |
|  | M3: | M2 + Cubic time | 6 | 23017 | 23057 | 0.08 | .773 |
| 2) | Selection of main effects | |  |  |  |  |  |
|  | M4: | M2 + Child gender | 6 | 22994 | 23034 | 23.38 | <.001 |
|  | M5: | M4 + Child age | 7 | 22995 | 23042 | 0.64 | .424 |
|  | M6: | M5 + Child attending care | 8 | 22950 | 23004 | 46.89 | <.001 |
|  | M7: | M6 + Other child(ren) | 9 | 22951 | 23011 | 1.06 | .303 |
|  | M8: | M7 + Parental MH | 10 | 22909 | 22976 | 43.86 | <.001 |
|  | M9: | M8 + Anyone vulnerable (medical condition) | 11 | 22909 | 22982 | 2.91 | .088 |
| 3) | Selection of interaction effects | |  |  |  |  |  |
|  | M10: | M9 + Child gender * (Linear & Quadratic time) | 13 | 22912 | 22999 | 0.87 | .648 |
|  | M11: | M9 + Child age *(Linear & Quadratic time) | 13 | 22905 | 22992 | 7.81 | .020 |
|  | M12: | M11 + Child attending care * (Linear & Quadratic time) | 15 | 22893 | 22993 | 16.14 | <.001 |
|  | M13: | M12 + Other child(ren) * (Linear & Quadratic time) | 17 | 22891 | 23005 | 5.54 | .063 |
|  | M14: | M12 + Parental MH * (Linear & Quadratic time) | 17 | 22895 | 23009 | 1.33 | .514 |
|  | M15: | M12 + Anyone vulnerable (medical condition) * (Linear & Quadratic time) | 17 | 22892 | 23006 | 4.43 | .109 |
| 4) | Final model [M16] | |  |  |  |  |  |
|  | M0 + Linear time + Quadratic time + Child gender + Child age + Child attending care + Parental MH + Child age *(Linear & Quadratic time) + Child attending care * (Linear & Quadratic time) | | 13 | 22893 | 22980 |  |  |

*Note.* Final model of the main analysis includes all effects identified as improving model fit in the previous steps of the main analysis.

Supplementary References

1. GOV.UK. Male and female populations. Published 2018. Accessed December 12, 2022. https://www.ethnicity-facts-figures.service.gov.uk/uk-population-by-ethnicity/demographics/male-and-female-populations/latest

2. Office for National Statistics. A01: Summary of labour market statistics. Published online November 15, 2022. Accessed December 12, 2022. https://www.ons.gov.uk/employmentandlabourmarket/peopleinwork/employmentandemployeetypes/datasets/summaryoflabourmarketstatistics

3. Clark. D. UK population 2020, by region. Published online November 7, 2022. Accessed December 12, 2022. https://www.statista.com/statistics/294729/uk-population-by-region/

4. Office for National Statistics. *Ethnic Group, England and Wales*.; 2022. Accessed December 12, 2022. https://www.ons.gov.uk/peoplepopulationandcommunity/culturalidentity/ethnicity/bulletins/ethnicgroupenglandandwales/census2021

5. Office for National Statistics. Average household income, UK. Published January 21, 2021. Accessed December 12, 2022. https://www.ons.gov.uk/peoplepopulationandcommunity/personalandhouseholdfinances/incomeandwealth/bulletins/householddisposableincomeandinequality/financialyear2020
